# Effect of orbital fat decompression on eyelid contour according to midpupil lid distance in graves’ ophthalmopathy

**DOI:** 10.1186/s12886-025-04435-8

**Published:** 2025-10-31

**Authors:** Yu-Wen Wang, Wei-Lun Huang, Shu-Lang Liao, Yi-Hsuan Wei

**Affiliations:** 1 Department of Ophthalmology, National Taiwan University Hospital, College of Medicine, National Taiwan University, Taipei, Taiwan; 2 Department of Ophthalmology, National Taiwan University Hospital Hsinchu Branch, Hsinchu, Taiwan; 3 Graduate Institute of Clinical Medicine, College of Medicine, National Taiwan University, Taipei, Taiwan; 4 College of Medicine, National Taiwan University, Taipei, Taiwan

**Keywords:** Eyelid contour, Eyelid retraction, Graves' ophthalmopathy, Midpupil lid distance, Orbital fat decompression

## Abstract

**Background:**

This study aimed to describe and compare eyelid contours before and after orbital fat decompression in patients with Graves’ ophthalmopathy (GO) by measuring their midpupil lid distances (MPLDs).

**Methods:**

A retrospective comparative study of patients with GO who underwent orbital fat decompression was performed. Standard digital images of primary gaze were analyzed using a self-designed software. Radial MPLDs of the upper and lower eyelids were measured from 0° (nasal) to 180° (temporal) with 15° spacing. Pre- and postoperative MPLDs were compared and the relationship between MPLD changes and Hertel exophthalmometric value changes were analyzed. Eyelid contour symmetries were assessed using the nasal-to-temporal MPLD ratios.

**Results:**

A total of 31 eyes from 17 patients and 25 eyes from 17 normal participants (control) were included. The mean postoperative Hertel value change was 3.8 ± 1.1 mm. After orbital fat decompression, MPLDs in the lower eyelids decreased, more significantly on the nasal sectors (nasal 30°, 45°, 60°, 75°, and 90°, *p* = 0.030, 0.024, 0.036, 0.040, and 0.042, respectively). Conversely, MPLDs slightly increased in the upper eyelids but was not statistically significant. Changes in MPLDs did not correlate with the extent of proptosis reduction.

**Conclusions:**

Orbital fat decompression effectively improves lower eyelid retraction in patients with GO, especially on the nasal sectors. This effect is independent from the amount of proptosis reduction. Upper eyelid contour remains unchanged after the surgery.

## Background

Graves’ ophthalmopathy (GO) is an autoimmune inflammatory disorder that encompasses various ophthalmologic manifestations, with eyelid retraction and proptosis being the most common [1]. The pathogenesis of GO is characterized by activation of orbital fibroblasts driven in part by autoreactive T-cell–mediated responses and T-cell–fibroblast crosstalk, leading to de novo adipogenesis, increased production of hyaluronan, and myofibroblast differentiation. These pathological changes lead to extraocular muscle enlargement and orbital fat expansion within the limited space of the bony orbit, elucidating most of the clinical manifestations of GO [2–6]. While active phase of GO can be managed with immunosuppressive and biologic agents [7], rehabilitative surgeries are often required for residual manifestation in the stable phase [7–9].

Orbital decompression is typically the first and fundamental stage for surgical rehabilitation of GO, with the aim of alleviating symptoms by enlarging the restricted orbital space or by removing the orbital content. Traditionally, this is achieved by removing one or more orbital walls, known as orbital bony decompression. However, this procedure is associated with postoperative complications such as strabismus, diplopia, infraorbital anesthesia, hypoglobus, sinusitis, and rarely, cerebrospinal fluid leak [10, 11]. The risk of postoperative diplopia is technique dependent: deep lateral wall decompression and fat decompression are generally associated with low rates of new-onset primary-gaze diplopia, whereas inferomedial and balanced wall approaches carry higher rates [12]. Against this backdrop, orbital fat decompression, which removes only the intraorbital fat and preserves orbital wall integrity, has gained increasing popularity in patients with mild-to-moderate proptosis and no significant orbital myopathy or compressive neuropathy in recent years. It has long-term efficacy in correcting mild-to-moderate disfiguring proptosis, with a lower rate of complications and without the need for secondary decompression procedures, in a more predictable manner [13–15].

Orbital decompression reportedly not only reduces proptosis but also improves eyelid position [16–18]. However, most of previous studies employed orbital bony decompression as the primary procedure, and the effect of orbital fat decompression on eyelid position remains largely unknown. Furthermore, eyelid position is commonly assessed with the margin reflex distance (MRD), which is the vertical distance between the central corneal light reflex and the eyelid margin. However, GO may exhibit eyelid contour deformities such as lateral lid flare and flat appearance, which are not reflected in MRD measurements. To address the changes in eyelid contour, multiple radial midpupil lid distance (MPLD) measurement, a simple and effective method to analyze eyelid contour, was first described by Milbratz et al. [19] and has been increasingly used in recent years [20, 21]. Compared to the single vertical measurement of MRD, radial MPLD captures multiple angular points along the eyelid margin, enabling a more comprehensive assessment of contour changes and detection of localized eyelid contour deformities.

In this study, we described and compared the pre- and postoperative eyelid contours and their correlations to the extent of exophthalmos by measuring MPLDs in patients with GO undergoing orbital fat decompression.

## Methods

**Fig. 1:**
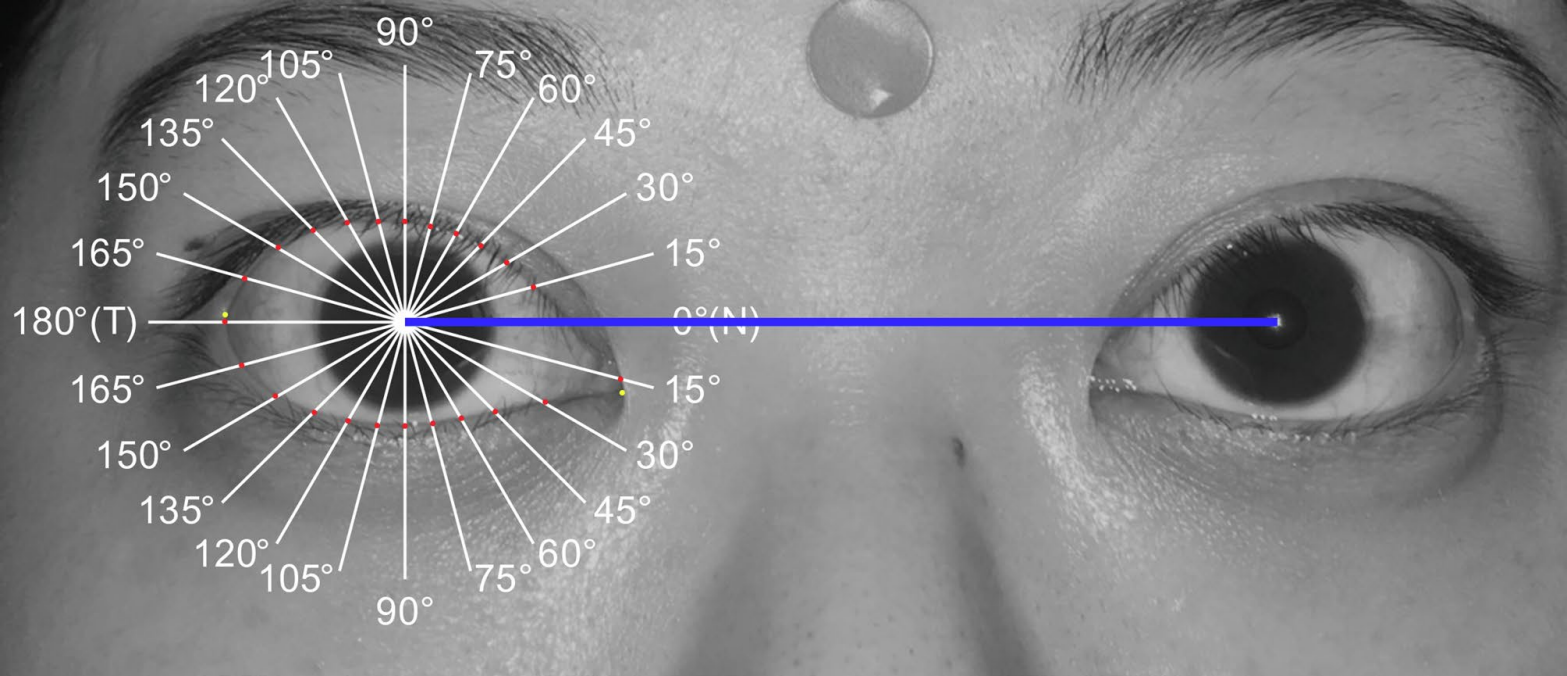
Multiple midpupil lid lines (15°). The blue line represents the interpupillary line. N, nasal sector; T, temporal sector

This retrospective comparative study was approved prospectively by the National Taiwan University Hospital Research Ethics Committee (IRB No. 202307048RINB). Due to the retrospective design, the requirement for informed consent for chart review and image analysis was waived. Written informed consent for publication of the facial photograph shown in Fig. 1 was obtained from that patient. The study complies with the tenets of the Declaration of Helsinki.

### Participant enrollment

All adult patients with the diagnosis of GO who received orbital fat decompression at the Department of Ophthalmology of National Taiwan University Hospital during January 2016 to December 2017 were retrospectively reviewed. Prior to the surgery, the patients were in euthyroid status for more than 3 months, and had stable Hertel exophthalmometric readings for more than 6 months. Preoperative orbital CT or MRI was performed in every patient to assess the relative contributions of orbital fat expansion versus extraocular muscle enlargement to proptosis. Patients were selected for orbital fat decompression alone if they demonstrated mild-to-moderate proptosis (preoperative Hertel < 24 mm), no significant extraocular muscle enlargement, and no evidence of compressive optic neuropathy [9]. We excluded those who had a history of eyelid, orbital, or facial surgery, trauma or inflammation leading to cicatrization. Baseline eyelid contour features (including the presence of temporal flare) were assessed qualitatively on standardized frontal photographs; no specific threshold was used for exclusion. Patients with significant strabismus leading to difficulty maintaining the primary gaze position, and those with incomplete photographs for eyelid contour analysis, were also excluded. Patient demographics, pre- and postoperative standard digital images of both eyes, pre- and postoperative Hertel exophthalmometric values, and surgical data including resected orbital fat volumes were collected. Normal controls that had no GO or other orbital or eyelid diseases in at least one eye who received eye photography at the outpatient clinic in the same timeframe were included.

### Surgical techniques

All participants with Graves’ ophthalmopathy underwent orbital fat decompression via the transforniceal approach. Initially, the surgical field was exposed by placing two 4 − 0 silk traction sutures through the center of the lower eyelid and inferior edge of bulbar conjunctiva to pull the eyelid downward and conjunctiva upward, respectively. Next, a subconjunctival incision was made through the inferior fornix, followed by separation and dissection of the lower eyelid retractors. Prolapsing extraconal and intraconal orbital fat were resected in a balanced manner from the medial and lateral compartments from the orbital margin deep into the intraconal apex after blunt dissection to prevent the potential damage to the surrounding tissues including extraocular muscles. Very little fat was removed from the central-inferior orbital compartment, intentionally avoiding the region close to the ciliary ganglion to minimize the risk of pupillary dysfunction. In addition, gentle posterior pressure was applied to the globe intraoperatively to redistribute orbital fat. Meticulous cauterization over the resected margin of the orbital fat was performed to prevent retrobulbar hemorrhage. The desired volume of orbital fat was measured intraoperatively using a 5- or 10-mL syringe. The conjunctival wound was then closed with 6 − 0 polyglactin interrupted sutures. Finally, a Frost traction suture was applied at the center of the lower eyelid and secured to the forehead using surgical tape for 3 days after the surgery. All surgeries were performed by the same surgeon (SLL).

### Image acquisition and eyelid contour analysis

All patients were photographed within 2 months preoperatively and 3 months postoperatively. Both the patients and normal participants (controls) were asked to sit and fixate on a distant target, and then photographed with flash in the primary position of gaze using a digital camera (Canon DIGITAL IXUS 950 IS; Canon Inc., Tokyo, Japan) positioned in the frontal plane at pupil height and at an approximate distance of 1.0 m to minimize distortion. Length was calibrated using a 9-mm round sticker attached to the middle of the forehead. All photos were analyzed using a self-designed software. This software automatically recognized the pupil center and drew the horizontal interpupillary line at 0° and 180°. We defined the nasal sector of the eyelid fissure as 0°, and the temporal sector as 180°. Next, a traditional 90° vertical line (MRD) was drawn, along with five radial lines spaced 15° apart from the vertical line, in both the temporal sectors (105°, 120°, 135°, 150°, and 165°) and nasal sectors (75°, 60°, 45°, 30°, and 15°) of the eyelid fissure (Fig. 1). The observer marked the intersections of the radial lines on both the upper and lower eyelid margin edges, and the software automatically calculated the radial MPLD lengths. All MPLD measurements were conducted by the same observer (WLH) to ensure methodological consistency. To analyze eyelid contour symmetries, we calculated the nasal-to-temporal MPLD ratios, which share the same angles relative to the midline, including 75°/105°, 60°/120°, 45°/135°, and 30°/150°. Considering the complexity and variability of the eyelid contour near both canthi, MPLDs at 0°, 15°, 165°, and 180° were excluded from our analysis.

### Statistical analysis

Statistical analyses were conducted using commercially available software (Microsoft Office Excel 2017, Redmond, WA, USA and SPSS version 29.0, SPSS Inc., Chicago, IL, USA). Analyses of MPLD changes after orbital fat decompression were performed using the Wilcoxon matched-pairs signed rank test. Correlations between MPLD changes in the upper and lower eyelid and Hertel value changes after orbital fat decompression were determined using Spearman’s rank-order correlation. Intergroup analyses of nasal-to-temporal MPLD ratios were performed using the Mann-Whitney U test. A *p* value less than 0.05 was considered statistically significant.

## Results

This study included 31 eyes from 17 eligible patients and 25 eyes from 17 normal controls. Demographic and surgical data of the patients were summarized in Table 1. The mean age was 40.1 ± 11.7 years. Most of the patients were female (*n* = 15, 88%), and only 4 (23%) were current smokers. The mean Hertel values before and after orbital fat decompression were 20.8 ± 1.3 and 17.0 ± 1.0 mm, respectively. The mean postoperative Hertel value change was 3.8 ± 1.1 mm (2.0–6.0 mm), and the mean volume of resected orbital fat was 4.1 ± 1.0 mL (1.5–5.8 mL). No intraoperative or postoperative complications, such as retrobulbar hemorrhage, orbital infection, wound dehiscence, cerebrospinal fluid leak, vision-threatening events, or new-onset diplopia were observed.

**Table 1:** Demographics and surgical data of participants with Graves’ ophthalmopathy

Variables	Mean ± Standard Deviation (range) or Number (%)
Age (years)	40.1 ± 11.7 (23–62)
Sex	
Female	15 (88%)
Male	2 (12%)
Smoking status	
Current smoker	4 (23%)
Previous smoker (quitted)	2 (12%)
Never-smoker	11 (65%)
Hertel exophthalmometric value (mm)	
Preoperative	20.8 ± 1.3 (18.5–23.0)
Postoperative	17.0 ± 1.0 (15.5–18.5)
Reduction	3.8 ± 1.1 (2.0–6.0)
Resected orbital fat volume (mL)	4.1 ± 1.0 (1.5–5.8)

**Fig. 2:**
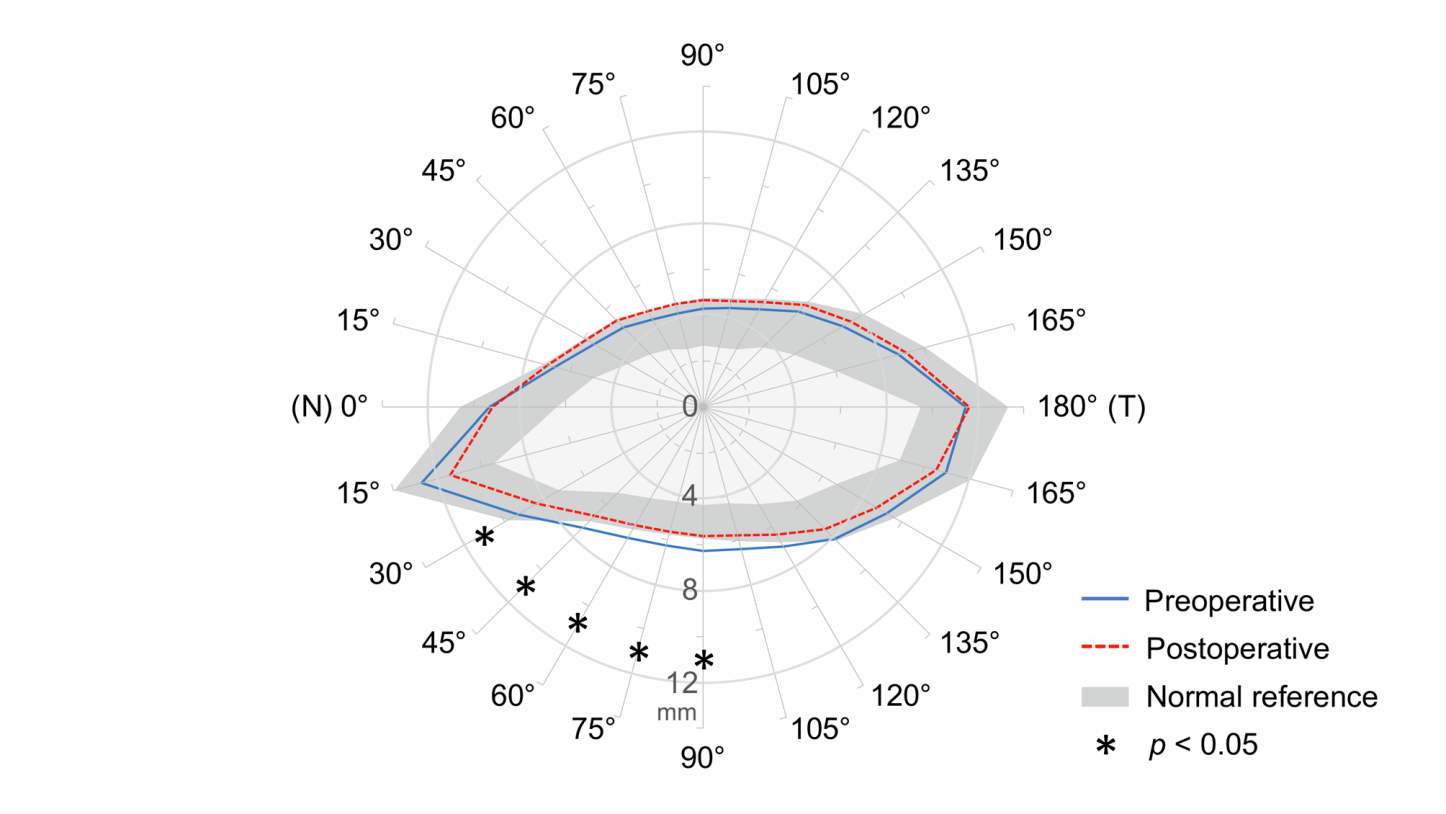
The average pre- and postoperative eyelid contour in GO patients undergoing orbital fat decompression displayed on a polar plot. The gray band represents the normal reference based on the 5th and 95th percentiles of MPLDs in the control group. N, nasal sector; T, temporal sector

The average pre- and postoperative eyelid contours and the normal reference based on the 5th and 95th percentile of MPLDs in the control group were displayed in Fig. 2. After surgery, there were opposite changes in the upper and lower eyelid. In the upper eyelid, MPLDs were slightly increased at all angles, however, none of these changes reached statistical significance (sector-wise *p* values are reported in Table 2; all *p* > 0.05). Conversely, MPLDs in the lower eyelid decreased, especially on the nasal sectors (nasal 30°, 45°, 60°, 75°, and 90°), and the differences were statistically significant (*p* = 0.030, 0.024, 0.036, 0.040, and 0.042, respectively) (Table 2). Further correlation analysis between surgically induced MPLD changes and Hertel value changes showed no significant correlation in both upper and lower eyelids (Table 3).

**Table 2:** Changes in MPLDs after orbital fat decompression in the upper and lower eyelid

MPLDs (mm)				
	Preoperative	Postoperative		
Angle	Mean ± SD	Mean ± SD	Mean Difference ± SD	*p*
**Upper eyelid**				
Nasal 30°	5.49 ± 1.35	5.88 ± 1.31	0.39 ± 1.83	0.176
Nasal 45°	4.91 ± 1.30	5.33 ± 1.33	0.42 ± 1.82	0.217
Nasal 60°	4.41 ± 1.21	4.83 ± 1.31	0.42 ± 1.78	0.248
Nasal 75°	4.23 ± 1.16	4.65 ± 1.29	0.41 ± 1.73	0.272
Median 90°	4.29 ± 1.17	4.66 ± 1.26	0.37 ± 1.75	0.327
Temporal 105°	4.47 ± 1.22	4.78 ± 1.25	0.31 ± 1.81	0.445
Temporal 120°	4.92 ± 1.34	5.28 ± 1.30	0.35 ± 1.93	0.357
Temporal 135°	5.89 ± 1.56	6.30 ± 1.43	0.40 ± 2.13	0.367
Temporal 150°	7.05 ± 1.74	7.45 ± 1.56	0.40 ± 2.45	0.433
**Lower eyelid**				
Nasal 30°	9.36 ± 1.80	8.40 ± 1.74	−0.95 ± 2.10	0.030*
Nasal 45°	7.44 ± 1.46	6.69 ± 1.30	−0.75 ± 1.69	0.024*
Nasal 60°	6.58 ± 1.28	5.94 ± 1.16	−0.64 ± 1.53	0.036*
Nasal 75°	6.23 ± 1.19	5.62 ± 1.11	−0.62 ± 1.45	0.040*
Median 90°	6.27 ± 1.16	5.62 ± 1.10	−0.65 ± 1.47	0.042*
Temporal 105°	6.41 ± 1.22	5.78 ± 1.16	−0.62 ± 1.57	0.057
Temporal 120°	7.02 ± 1.32	6.41 ± 1.30	−0.61 ± 1.73	0.078
Temporal 135°	8.13 ± 1.46	7.52 ± 1.52	−0.61 ± 2.02	0.117
Temporal 150°	9.26 ± 1.37	8.76 ± 1.50	−0.50 ± 1.94	0.153

*MPLDs *Midpupil lid distances, *SD *Standard deviation

* Statistically significant at *p* < 0.05

**Table 3:** Correlation between MPLD changes in the upper and lower eyelid and Hertel value changes after orbital fat decompression

MPLD changes versus Hertel value changes		
	Spearman’s ρ	*p*
**Upper eyelid**		
Nasal 30°	0.167	0.368
Nasal 45°	0.174	0.349
Nasal 60°	0.153	0.410
Nasal 75°	0.132	0.478
Median 90°	0.135	0.469
Temporal 105°	0.114	0.542
Temporal 120°	0.124	0.507
Temporal 135°	0.139	0.455
Temporal 150°	0.135	0.468
**Lower eyelid**		
Nasal 30°	0.131	0.482
Nasal 45°	0.143	0.443
Nasal 60°	0.215	0.245
Nasal 75°	0.175	0.346
Median 90°	0.168	0.367
Temporal 105°	0.149	0.423
Temporal 120°	0.120	0.522
Temporal 135°	0.098	0.600
Temporal 150°	0.153	0.412

*MPLDs* Midpupil lid distances

Regarding eyelid contour symmetry, on qualitative review, no patients exhibited obvious outliers or special contour features (e.g., pronounced temporal flare). Quantitatively, the nasal-to-temporal MPLD ratios in both the upper and lower eyelids were similar in the preoperative GO group and the control group. Postoperatively, such ratios in both the upper and lower eyelids did not show any statistically significant changes (*p* > 0.05 for all pairs) (Table 4).

**Table 4:** Comparison of nasal-to-temporal MPLD ratios in the control group and GO patients before and after orbital fat decompression

	Nasal-to-temporal MPLD ratios			*p* values	
	Control	Pre-op GO	Post-op GO	Pre-op vs. Control	Pre-op vs. Post-op
**Upper eyelid**					
N 75°/T 105°	0.957 ± 0.030	0.948 ± 0.037	0.971 ± 0.049	0.216	0.060
N 60°/T 120°	0.920 ± 0.061	0.900 ± 0.070	0.914 ± 0.074	0.185	0.481
N 45°/T 135°	0.862 ± 0.081	0.845 ± 0.118	0.851 ± 0.095	0.315	0.784
N 30°/T 150°	0.801 ± 0.091	0.795 ± 0.136	0.795 ± 0.095	0.473	0.544
ΣN/Σ T	0.874 ± 0.068	0.861 ± 0.096	0.871 ± 0.079	0.327	0.505
**Lower eyelid**					
N 75°/T 105°	0.971 ± 0.030	0.973 ± 0.027	0.972 ± 0.033	0.748	0.710
N 60°/T 120°	0.931 ± 0.057	0.937 ± 0.052	0.930 ± 0.049	0.463	0.456
N 45°/T 135°	0.902 ± 0.076	0.916 ± 0.086	0.894 ± 0.078	0.515	0.433
N 30°/T 150°	1.029 ± 0.117	1.007 ± 0.105	0.957 ± 0.089	0.650	0.052
ΣN/Σ T	0.962 ± 0.072	0.959 ± 0.067	0.937 ± 0.061	0.850	0.196

MPLD ratios are given in mean ± standard deviation (SD)

*GO* Graves’ ophthalmopathy, *MPLDs *Midpupil lid distances, *N *Nasal sector, *Pre-op *Preoperative, *Post-op *P ostoperative, *T *Temporal sector

## Discussion

Eyelid retraction is the most common and characteristic manifestation of GO. Various theories have been proposed to explain its etiology. Mechanisms such as inflammation, enlargement, and subsequent fibrosis of the extraocular muscles and eyelid retractors might contribute to eyelid retraction. Davies et al. retrospectively reviewed the computed tomography scans of orbits from 50 consecutive patients with unilateral GO-related upper eyelid retraction. They found that levator palpebrae superioris (LPS) was larger on the retracted side than on the nonretracted side in over 85% of patients; hence, inflammation, enlargement, and possible fibrosis of LPS primarily drove upper eyelid retraction in GO [22]. As for the lower eyelid, anatomically, the lower eyelid retractor is inherently connected to the inferior oblique and rectus muscle via the capsulopalpebral fascia, and alterations in this dynamic system affect the lower eyelid position. Inferior rectus muscle inflammation and enlargement, which are often seen in GO, can thus retract the lower eyelid because of excessive tension [23–25].

Recent studies have reported that the lower but not upper eyelid retraction reduce after orbital bony decompression, with or without combined fat removal [17, 26]. However, studies addressing the effect of solely orbital fat decompression on eyelid position and contour remain scarce. In the present study, orbital fat decompression alone also had a positive therapeutic effect on lower eyelid retraction, particularly on the nasal sectors. Conversely, upper eyelid retraction did not improve postoperatively. In fact, a statistically insignificant elevation was observed in the upper eyelid position. One potential explanation for this observation is that we primarily remove fat from the inferior part of the orbit, leading to a subtle downward displacement of the eyeball. However, given that this effect does not reach statistical significance, we did not perceive any noticeable difference in the upper eyelid position postoperatively. The specific therapeutic effect on the nasal sectors of the lower eyelid may result from the unique anatomic features of the lower eyelid retractor. Similar to the upper eyelid retractor, the lower eyelid retractor consists of medial and lateral horns, with the latter being larger [27]. This anatomical feature contributes to a stronger lateral force exerted on the lower eyelid, making the improvement of lower eyelid retraction on the temporal side more challenging.

Another frequently cited theory for the mechanism of eyelid retraction in GO is the mechanical effect of proptosis. Protrusion of the globes displaces the eyelids toward the equator and widens the palpebral fissure, thereby mechanically exacerbating the eyelid retraction [16, 25, 28]. Nevertheless, in our cohort, which included only patients with mild-to-moderate proptosis (Hertel < 24 mm), changes in MPLDs did not correlate with the amount of proptosis reduction in the upper or lower eyelid after surgery. This suggests reversal of the mechanical effects of proptosis is not the primary mechanism by which orbital fat decompression improves lower eyelid retraction. Instead, it may be attributed to the dissection of the lower eyelid retractors intraoperatively, effectively weakening the force of retraction. In addition, we routinely apply a Frost traction suture at the center of the lower eyelid and tape it to the forehead to keep the eyelid on stretch for 3 days after surgery. This step prevents the dissected lower eyelid retractor from adhesion and lowers the insertion site during healing, consequently reducing the force of retraction on the lower eyelid. Our finding differs from Cho’s study, which showed a significant correlation between the amount of proptosis reduction and improvement in lower eyelid position at 3 months after medial, lateral, or balanced orbital decompression (correlation = 0.17, *p* = 0.037) [16]. This discrepancy might arise from the difference in surgical procedures. Their study employed orbital bony decompression as the primary procedure, with balanced two-wall decompression in most of their cases (75.2%). Removing one or more orbital walls increases more orbital volume, thereby reducing proptosis more substantially than removing intraorbital fat alone. The mechanism affecting eyelid position in their study predominantly involves reversing the mechanical effect of proptosis. Moreover, we believe that our findings are strengthened by the measurement of multiple radial MPLDs from different eyelid angles, as opposed to solely assessing the inferior scleral show at 90°, as conducted in their study.

Temporal flare is a frequently observed characteristic in GO-related eyelid retraction, indicating a more pronounced retraction laterally than medially, leading to an anomalous upper eyelid contour. However, in our study, there were no statistically significant differences in the nasal-to-temporal MPLD ratios in both the upper and lower eyelid between the preoperative GO group and the control group. In other words, our patients did not exhibit significantly asymmetric eyelid contours. After surgery, the ratios in both the upper and lower eyelid showed no significant changes. This result is consistent with a previous study reporting that normal eyelid contours were only minimally affected, whereas the upper eyelid contour normalized in 40% of patients exhibiting temporal flare after undergoing orbital bony decompression [29]. Future studies including patients with severe temporal flare are needed to investigate the potential influence of orbital fat decompression on asymmetric eyelid contours.

Conventionally, surgical rehabilitation for GO involves a staged approach, with the initial step being orbital decompression, followed by strabismus surgery and then eyelid reposition surgery as necessary [30]. However, performing eyelid reposition surgery during orbital decompression is increasingly advocated. Ben Simon GJ et al. showed that surgery for upper eyelid retraction in patients with GO yielded comparable outcomes regardless of whether it was performed simultaneously with orbital bony decompression or at a later stage [31]. In our investigation, we found a positive therapeutic effect of orbital fat decompression on lower eyelid retraction, while noting no significant change in the upper eyelid. These findings support the feasibility of combining upper eyelid retraction surgery with orbital fat decompression. However, for correcting lower eyelid retraction, maintaining a staged approach is recommended, given that the lower eyelid may undergo changes during orbital fat decompression, particularly on the nasal sectors.

To our knowledge, reports regarding the pre- and postoperative eyelid contours in patients with GO undergoing solely orbital fat decompression remain unavailable. This study is the first to present the positive therapeutic effect of solely orbital fat decompression on lower eyelid retraction in patients with GO. However, our study has several limitations. First, it was retrospective in nature with a relatively limited sample size. Second, although MRD-1 and MRD-2 were inherently captured within our MPLD system at 90° (upper median 90° ≈ MRD-1; lower median 90° ≈ MRD-2), we did not report these values separately as conventional MRD-1 and MRD-2 measurements. In addition, orbital fat decompression is mainly indicated for patients with mild-to-moderate proptosis (preoperative Hertel value < 24 mm) and without significant preoperative diplopia [9]. Thus, the study findings may not be applicable to patients with severe proptosis. In addition, our cohort did not include patients with clinically significant temporal flare or marked baseline eyelid asymmetry, and the results should not be generalized to those populations. Future studies will incorporate continuous contour modeling and will prospectively enroll patients with temporal flare to enable pre-specified subgroup analyses. Finally, for patients who present with strabismus or diplopia preoperatively, many of them may require strabismus surgery in the future, potentially influencing the final eyelid position. Therefore, surgeons should exercise caution and carefully consider this factor during operative planning for these patients.

## Conclusion

In summary, this study provides evidence that orbital fat decompression alone effectively improves lower eyelid retraction in patients with GO, especially on the nasal sectors, and this effect does not correlate with the amount of proptosis reduction. Although the upper eyelid position is also affected by orbital fat decompression, this effect is insignificant and does not correlate with proptosis reduction. Given these findings, combining correction surgery for upper eyelid retraction with orbital fat decompression may be a viable approach to reduce the overall number of surgical procedures and accelerate surgical rehabilitation.

## Data Availability

The datasets used and/or analyzed during the present study are available from the corresponding author on reasonable request.
